# Exploring Factors of Diagnostic Timing Among Black Autistic Youth

**DOI:** 10.1007/s10803-024-06283-9

**Published:** 2024-03-21

**Authors:** Harlee Onovbiona, Lauren Quetsch, Emily-Anne Del Rosario

**Affiliations:** Department of Psychological Science, University of Arkansas, 306 Memorial Hall, Fayetteville, AR 72701 USA

**Keywords:** Autism spectrum disorder, Early identification, Barriers, Diagnosis, Black families

## Abstract

The goal of the present study was to compare profiles among Black families of autistic youth who were identified Early (≤ 2 years of age), Mid (age 3 or 4), and Delayed (≥ 5 years of age) to better identify the characteristics that contribute to early ASD identification and delayed ASD identification. Black caregivers with autistic youth (*N* = 101) were divided into Early (*N* = 34), Mid (*N* = 39), and Delayed (*N* = 28) groups and compared on (a) the age at which signs of autism signs were first noticed, (b) wait times, (c) previous misdiagnoses rates, and (d) racial barriers experienced during the diagnostic process. The results revealed differences between the diagnostic profiles. Specifically, (a) Delayed families noticed the first signs of autism significantly later, (b) Early families had significantly smaller wait times between age of noticing signs of autism and age of receiving the diagnosis, (c) the odds of receiving a later or delayed autism diagnosis was nearly three times higher for caregivers who reported receiving a misdiagnosis, and (d) there were no significant differences in racial barriers experienced between Early, Mid, and Delayed families. Challenges in receiving a timely diagnosis remain for some Black autistic youth. To improve early identification for Black autistic youth who are at risk for receiving delayed diagnostic care, further research should examine factors and practices that improve autism knowledge among professionals and caregivers, enhance assessment practices, and integrate culturally responsive practices into assessment and screening procedures.

Autism spectrum disorder^1^ is a neurodevelopmental condition which presents as differences in social communication and interactions as well as restricted, repetitive behavior patterns, interests, or activities (American Psychological Association, 2022). Signs of autism can be spotted as early as 12 months of age; however, many children fail to receive a formal diagnosis until the age of 4 years (Baio et al., 2018; Elder et al., 2017). While the Center for Disease Control and Prevention (CDC) currently reports higher autism prevalence rates among Black children compared to white peers (Maenner et al., 2023), Black autistic youth have historically been diagnosed nearly 3 years later than the national average (Constantino et al., 2020). These delayed diagnoses reduce access to early intervention services and optimal care for Black youth (Constantino et al., 2020; Pierce et al., 2016; Steinbrenner et al., 2022). As a result of this public health crisis, greater research and clinical efforts have been made to understand why this diagnostic gap exists and how to close it (Reyes et al., 2024; Weitlauf et al., 2022).

Prior literature has attributed delays in diagnostic timing to limited knowledge of autism characteristics among primary care providers and systemic factors. Black caregivers of autistic youth have stated concerns that their primary care providers poor competency in identifying autism symptomology impacted delays in receiving a timely diagnosis (Fisher et al., 2022). This finding is corroborated by a previous systematic review which found that healthcare professionals reported moderate levels of autism competency and self-efficacy and indicated poor access to trainings (Azim et al., 2020; Corden et al., 2021). Poor autism competency and awareness can perpetuate under-identification and misdiagnoses in the Black community (Aylward et al., 2021; Mandell et al., 2007; Shi et al., 2021). However, these misdiagnoses have also been attributed to provider racial biases such that disruptive behavior disorders are more likely to be considered in Black children than neurodevelopmental conditions (Durkin et al., 2017). Taken together, many medical providers have adopted a “wait and see” approach which encourages caregivers to wait until maturation or significant changes occur in their child to receive a diagnosis (Fisher et al., 2022; Smith-Young et al., 2020; Weitlauf et al., 2023).

Unfortunately, this strategy often minimizes caregiver concerns. Indeed, previous research also highlighted that providers and professionals have neglected the values, needs, and concerns of Black families (Lovelace et al., 2018). In several qualitative studies examining the experiences of Black caregivers of autistic youth as they navigate the diagnostic process, providers and professionals have been described as “unfamiliar with cultural differences” (Weitlauf et al., 2023, p. 5) with minimal compassion and patience (Fisher et al., 2022; Lovelace et al., 2018; Onovbiona et al., 2023; Stahmer et al., 2019). Ultimately, provider racial biases, provider lack of knowledge about autism, and structural barriers embedded within the healthcare delivery system can further contribute to poor quality care and impede early identification among Black families, further minimizing timely entry into intervention (Dababnah et al., 2018; Drame et al., 2020; Fisher et al., 2022; Zwaigenbaum et al., 2015).

In addition to provider factors, caregivers also play a crucial role in the timing of their child’s diagnosis (Becerra-Culqui et al., 2018). Often, caregivers are the first to notice differences in their child’s behaviors, development, or language and are therefore tasked with being their child’s primary advocate to establish adequate care and support (Pearson et al., 2020; Weitlauf et al., 2023). It therefore stands to reason that if caregivers lack knowledge about autism or typical developmental trajectories, efforts to pursue testing may be curtailed—thus delaying diagnostic timing. For instance, in a study conducted by Donohue et al. (2019), Black parents of autistic youth (before their children were diagnosed with autism) reported more concerns with their children’s disruptive behaviors than concerns with a possible neurodevelopmental condition (i.e., an autism diagnosis). Similarly, Black caregivers of autistic youth note difficulties with limited knowledge about autism and poor perceptions about autism within their family and community (Dababnah et al., 2018; Drame et al., 2020; Onovbiona et al., 2023; Pearson & Meadan, 2018; Stahmer et al., 2019). In a study exploring the screening, diagnostic, and intervention process for Black families, one mother noted that, “We need to remove the stigma around getting help for our children” (Weitlauf et al., 2023, p. 6), suggesting that familial stigma may interfere with caregiver motivation to seek diagnostic support for their child.

Indeed, professional (e.g., poor autism competency, racial bias, “wait and see” approach) and familial (e.g., poor autism knowledge, perceived stigma) factors may hamper the experience of Black families of autistic youth seeking a timely diagnosis. Yet, research remains less clear on what factors and characteristics may explicitly contribute to early identification as compared to delayed identification of autism in Black youth. To fill this gap, the present study will explore differences among Black families of autistic youth who were identified early (Early ≤ 2 years), around the national average (Mid = 3–4 years), and late (Delayed ≥ 5 years). Specifically, we will examine differences in the diagnostic age profiles (Ealy, Mid, Delayed) for (a) the age at which first signs of autism were noticed, (b) the wait gap between age first signs of autism were noticed and the age the child received a diagnosis, (c) the frequency of receiving misdiagnoses, and (d) experienced racial barriers (e.g., stigma, racial microaggressions). The results of the study will inform how to better serve Black families of autistic youth to bolster equitable and early identification of autism.

## Method

### Participants

A total of 101 Black caregivers of autistic youth were recruited through the Simon Foundation’s Powering Autism Research (SPARK) database across the United States (SPARK Consortium, 2018). The term caregiver is used to represent an adult who has legal guardianship of the child, which may or may not include a biological parent. The SPARK team invited caregivers of autistic children/dependents who identify as Black or African American and were a parent or guardian of an autistic child between 10 months to 20 years of age. The current study is a secondary research question from a parent study (Onovbiona et al., 2023). Institutional Review Board approval was obtained (Onovbiona et al., 2023) prior to recruiting or consenting caregivers.

### Procedures

This project was conducted as part of a larger study examining the barriers, parental stress, and treatment perceptions among Black caregivers of autistic youth using a mixed-method approach (Onovbiona et al., 2023). Caregivers completed an online survey with measures assessing caregiver stress, perceived racial barriers, logistical barriers, treatment perceptions, and diagnostic information. For the purposes of the present study, only measures related to diagnostic timing and racial barriers experienced during the diagnostic process were explored. Average survey duration time was 30 min. All participants who completed the questionnaires were emailed a $75 gift card.

### Measures

#### Diagnostic Factors

Caregivers completed questions about the age at which first signs of autism were noticed in their child (“*How old was your child when a professional first suggested that your child might have autism?*”) and the age at which their child received a diagnosis. Age of diagnosis was categorized into the following groups: (a) Early ≤ 2 years, (b) Mid = 3–4 years, and (c) Delayed ≥ 5 years. Additional questions asked about who was the first to notice signs of autism (“*Who first noticed symptoms of autism in your child?*”).

#### Wait Time

To determine the wait time for families, a variable was calculated by subtracting the age the first signs of autism were noticed from the age at which the child received a diagnosis.

#### Misdiagnoses

To assess prior misdiagnoses, caregivers responded to the statement, “*My child was misdiagnosed previously*.” Caregivers who agreed were coded a “1” and caregivers who disagreed were coded a “0.”

#### Racial Barriers to Treatment Participation Scale (RBTPS)

The study team created a brief questionnaire, the Racial Barriers to Treatment Participation Scale (RBTPS), to assess family experiences of racial barriers surrounding their interaction with healthcare providers. The measure was used in a previous study assessing the impact of barriers on treatment (redacted for blind review) and was found to predict parental stress. The racial barriers items were gathered from the literature on racial barriers experienced by Black families and Black families of autistic youth (e.g., stigma, perceived microaggressions; Broder-Fingert et al., 2020; Constantine, 2007; Pearson et al., 2018; Stahmer et al., 2019). The scale consists of 29 items scored on a 5-point Likert scale (0 = *Totally Disagree*, to 5 = *Totally Agree*). Three subscales measured three constructs related to racial barriers to treatment: Racial Microaggressions (10 items; e.g. ‘*My provider or therapist sometimes was insensitive about my cultural group when trying to understand or treat my concerns or issues.*’), Family and Community Beliefs (10 items; e.g., ‘*My family or friends blame me for child’s behavior problems.*’), and Treatment (9 items; e.g. ‘*It seemed like my provider was knowledgeable about the problems my child was facing.’*). Items from the microaggression subscale were taken from the Racial Microaggressions in Counseling Scale (Constantine, 2007). The summation of scores represented the total set of racial barriers to treatment participation. The RBTPS demonstrated good internal consistency (α = 0.90).

### Analytic Approach

IBM SPSS statistics software was used for data analyses. To assess differences in (a) the age at which signs of autism signs were first noticed, (b) the wait time, and (c) racial barriers between the diagnostic profiles (e.g., Early, Mid, Delayed), three separate one-way analyses of variances (ANOVAs) were conducted. The diagnostic profiles were entered as the independent variable. Age at which first autism signs were noticed, wait time, and racial barriers were all entered in as the dependent variables. An ordinal regression was conducted to examine the relation between the diagnostic profiles and previous experiences with misdiagnoses.

## Results

The overall sample included Black caregivers of autistic youth (*N* = 101) with autistic children between 10 months and 20 years of age. For the purposes of this study, the sample was divided into youth who received a diagnosis when they were 2 years or younger (Early;* N* = 34), around the national average (i.e., 3–4 years; Mid; *N* = 39), and at age 5 years or later (Delayed; *N* = 28). The majority of children were males (76.2%) and the caregivers were primarily mothers (90.1%). Over half of the sample reported an annual income of less than $65,000 (59.4%), while the rest of the sample reported an annual income of greater than $66,000 (40.6%). The mean age of receiving an autism diagnosis for the overall sample was 4 years (*SD* = 3.44). Across the diagnostic age profiles, caregivers were often the first to notice signs of autism (61.4%), followed by pediatricians (14.9%), school professionals (6.9%), friends and family (6.9%), psychiatrists (2.9%), other professionals (2.9%), child psychologists (1.9%), and pediatric neurologists (0.9%). A descriptive summary of the sample is presented in Table 1.

**Table 1:** Descriptive information for the diagnostic age profiles

Variable	Early (*n* = 34)	Mid (*n* = 39)	Delayed (*n* = 28)	Total (*N* = 101)
	*n*	*n*	*n*	*n* (%)
Location				
Rural	6	7	6	19 (18.8%)
Urban	15	14	10	39 (38.6%)
Suburban	13	18	12	43 (42.6%)
Caregiver age, in months *M*(*SD*)	38.35 (9.12)	39.08 (7.91)	42.11 (8.37)	39.65 (8.53)
Wait gap *M*(*SD*)	0.34 (0.47)	0.68 (0.62)	1.61 (2.30)	0.84 (1.40)
Age first signs *M*(*SD*)	2.045 (3.04)	2.97 (2.58)	6.86 (4.38)	3.76 (3.85)
Age of diagnosis *M(SD)*	1.69 (0.63)	3.23 (0.43)	8.46 (3.87)	4.163 (3.44)
Racial barriers *M*(*SD*)	59.97 (17.46)	68.28 (17.89)	67.57 (19.33)	65.29 (18.38)
Child gender				
Male	26	29	22	77 (76.2%)
Female	8	10	6	24 (23.8%)
Caregiver relation				
Mother	29	35	27	91 (90.1%)
Father	4	3	0	7 (6.93%)
Grandparent	1	1	1	3 (2.97%)
Income				
< $35,000	13	15	4	32 (31.7%)
$36,000–$65,000	10	10	8	28 (27.7%)
$66,000–$80,000	5	7	9	21 (20.8%)
Over $81,000	6	7	7	20 (19.8%)
Misdiagnosed				
Yes	6	7	13	26 (25.7%)
No	28	32	15	75 (74.3%)
First signs				
Pediatrician	7	5	3	15 (14.9%)
Me (caregiver)	20	26	16	62 (61.4%)
Psychiatrist	1	1	1	3 (2.9%)
Family/Friends	2	4	1	7 (6.9%)
Pediatric Neurologist	0	1	0	1 (0.9%)
School Staff	2	1	4	7 (6.9%)
Child Psychologist	0	0	2	2 (1.9%)
Other	1	1	1	3 (2.9%)

### First Autism Symptoms and Diagnostic Age Profiles

**Fig. 1:**
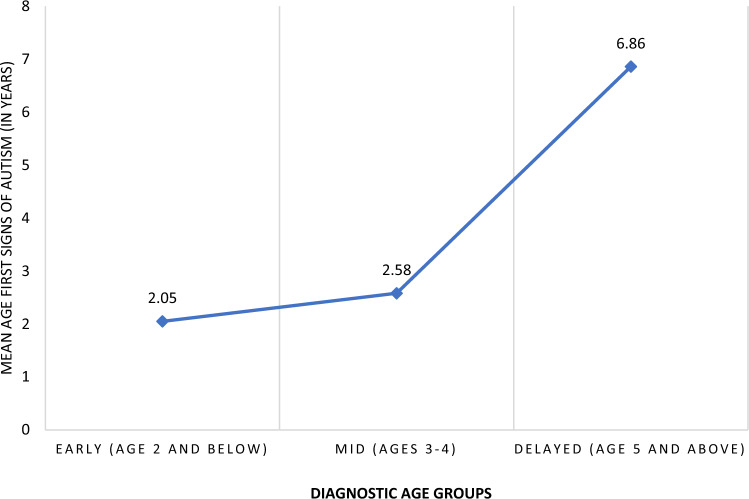
Mean age first signs of autism noticed by diagnostic age profiles

An ANOVA was conducted to examine differences in the age at which first signs of autism were noticed between diagnostic age profiles. Assumptions were not satisfactory for the test of homogeneity of variances; therefore, the Welch F-test was interpreted. The Welch test was significant, *F* (2, 56.15) = 12.148, *p* < 0.001 (Table 2). Games-Howell post-hoc comparisons revealed significant differences in the age first signs of autism were noticed between the Delayed and Early diagnostic profile (*t*(100) =  − 4.81; *p* < 0.001); and the Delayed and Mid diagnostic profile (*t*(100) =  − 3.89; *p* < 0.001). Those in the Delayed diagnostic profile noticed first signs of autism on average nearly three times later (*M* = 6.86, *SD* = 4.38) than those in the Early (*M* = 2.05, *SD* = 3.04) and Mid (*M* = 2.97, *SD* = 2.58) diagnostic profiles (Fig. 1).

**Table 2:** ANOVA and Welch tests comparing racial barriers, wait gap times, and age of first signs of autism

Variable	SS	df	MS	*F*	*p* value
Racial barriers					
Between	1456.95	2	728.47	2.21	.115
Wait gap times					
Between	25.28	2	12.640	6.642^a^	.003
Age first signs autism					
Between	389.09	2	194.54	12.148^a^	< .001

^a^Indicates that the Welch F-test was used

### Wait Times and Diagnostic Age Profiles

**Fig. 2:**
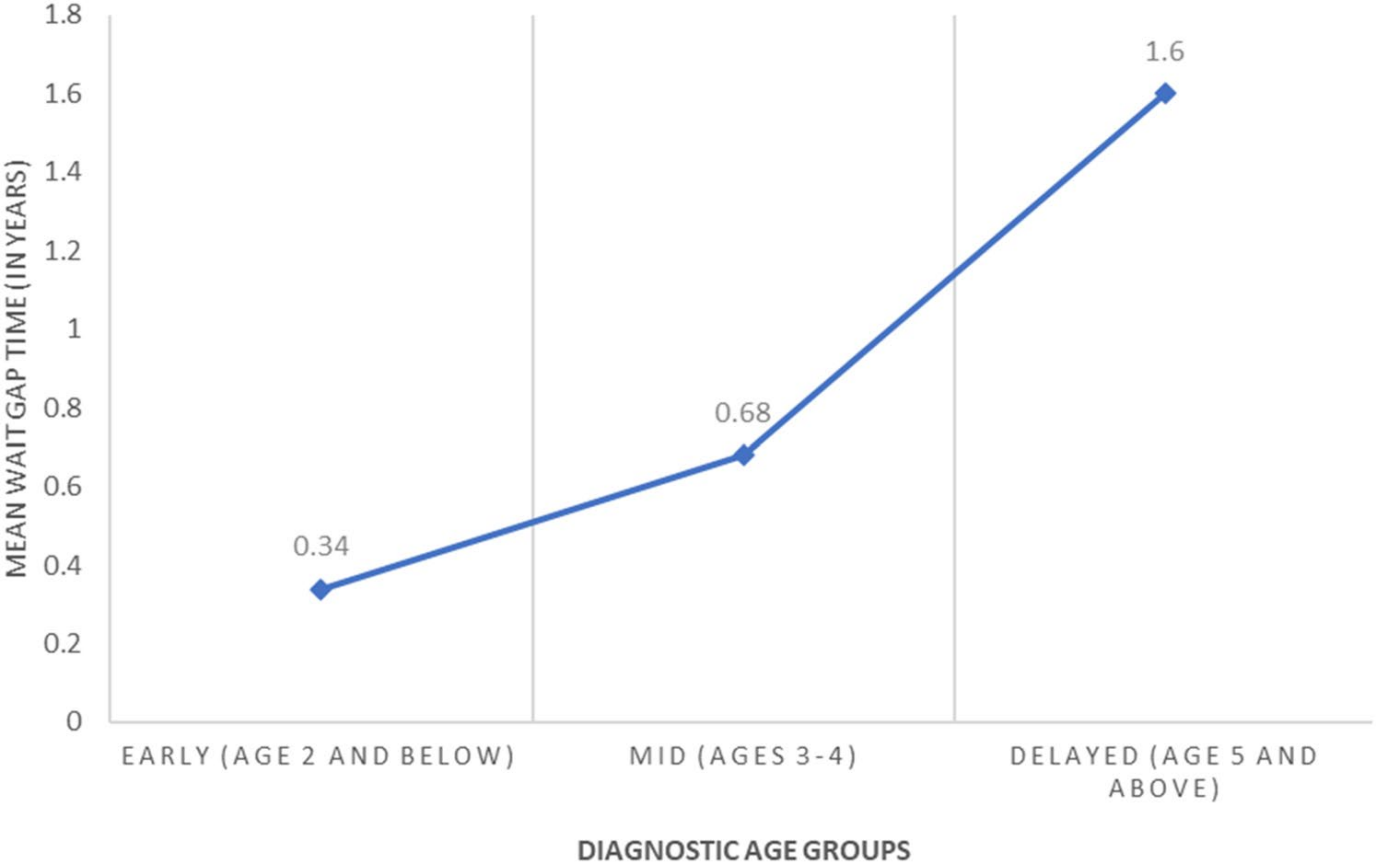
Mean wait gap times (age of diagnosis-age first signs noticed) by diagnostic age profiles

An additional ANOVA was conducted to examine differences in wait times between diagnostic age profiles. The variable failed to meet the test of homogeneity of variances, thus the Welch F-test was interpreted. The Welch test was significant, *F* (2, 52.30) = 6.642, *p* = 0.003 (Table 2). Games-Howell post-hoc comparisons revealed significant differences in wait times between the Delayed and Early diagnostic profile, *t*(100) = 1.26; *p* = 0.021; and the Early and Mid diagnostic profile, *t*(100) = 0.34; *p* = 0.029. Those in the Delayed diagnostic profile had significantly greater wait times (*M* = 1.6, *SD* = 2.30) compared to the Mid (*M* = 0.68, *SD* = 0.62) and Early (*M* = 0.34, *SD* = 0.47) diagnostic profiles (Fig. 2).

### Misdiagnoses and Diagnostic Age Profiles

**Fig. 3:**
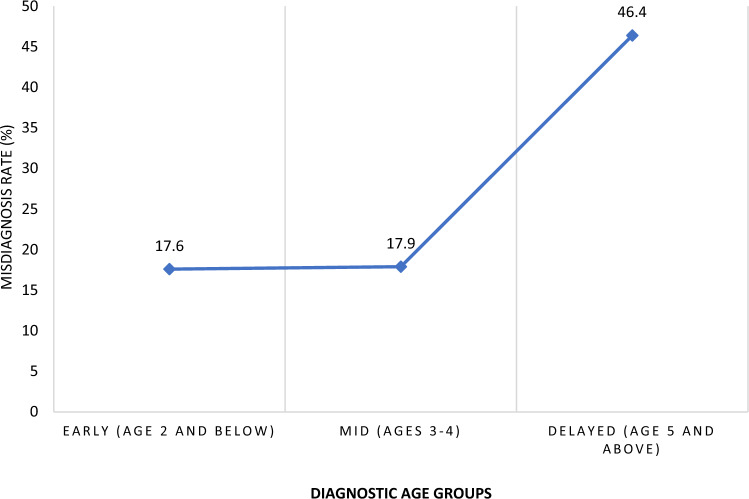
Autism misdiagnosis rates by diagnostic age profiles

An ordinal logistic regression was conducted to examine the relation between previous experiences with child misdiagnoses and the diagnostic age profiles. All assumptions were met, including goodness-of-fit. Diagnostic age levels were a significant predictor of previous experiences with child misdiagnoses, χ^2^(1) = 12.714, *p* < 0.012 (Table 3). The log odds of receiving a later or delayed autism diagnosis were higher on average for caregivers who reported receiving a misdiagnosis as compared to those who had not received a misdiagnosis previously (*β* = 0.753, SE = 0.311, *p* = 0.015). Caregivers in the Delayed diagnostic profile were nearly three times as likely to receive a misdiagnosis for their child (46.4%) than caregivers in the Mid (17.9%) and Early (17.6%) diagnostic profiles (Fig. 3).

**Table 3:** Ordinal regression analysis of diagnostic age profiles and previous experiences with misdiagnoses

Domain (predictor)	B	SE	Exp(B)	*p*	95% CI of Exp (B)	Chi-Square
1. Diagnostic age profile				.012		12.714
Misdiagnoses (Yes/No)	.753	.311	5.876	.015	.144–1.362	6.298

### Racial Barriers and Diagnostic Age Profiles

Lastly, an ANOVA was conducted to assess differences in caregiver reported racial barriers experienced between diagnostic age profiles. All assumptions were satisfactory. Caregivers in the Delayed (*M* = 67.57, *SD* = 19.33) and Mid (*M* = 68.28, *SD* = 17.89) diagnostic profiles exhibited greater racial barriers than caregivers in the Early diagnostic profile (*M* = 59.97, *SD* = 17.46). However, the ANOVA revealed no significant group differences in racial barriers, *F* (2, 98) = 2.21, *p* = 0.115 (Table 2). For the Racial Microaggression subscale, the most common racial barrier endorsed was in response to the following item: *Provider sometimes seemed unaware of the realities of race and racism (e.g., “When I look at you, I don’t see color.” Or “Everyone can succeed in this society, if they work hard enough.”*). 17.6% caregivers in the Early group, 33.3% of caregivers in the Mid group, and 29.6% of caregivers in the Delayed group agreed with this statement. Within the Family and Community Beliefs subscale, 81% of caregivers indicated that they do not trust health care professionals (85% of caregivers within the Early group, 74% of caregivers within the Mid group, and 82% of caregivers within the Delayed group). Lastly, within the Treatment subscale, nearly 80% of caregivers expressed feeling like their provider wasn’t knowledgeable (82% Early, 74% Mid, 71% Delayed) and indicated not understanding the language their providers used (70% Early, 69% Mid, 96% Delayed).

## Discussion

Previous findings have shed light on the barriers experienced by Black families of autistic youth as they seek an autism diagnosis for their child (e.g., differential treatment from providers, discrimination, poorer access to services, stigma, delayed diagnostic timing). Yet, there have been limited inquiries into characteristics that differentiate Black families who are diagnosed earlier, within the national average range, and later in life (Constantino et al., 2020; Onovbiona et al., 2023; Stahmer et al., 2019; Weitlauf et al., 2023). The findings of the present study revealed notable differences between Black families of autistic youth diagnosed at 2 years of age or before (Early), between the ages of 3 and 4 (Mid), and at or after the age of 5 (Delayed). In particular, the findings demonstrated that within a sample of geographically, socioeconomic, and ethnically diverse Black families, (a) Delayed families noticed the first signs of autism significantly later than the Mid and Early families, (b) Early families had significantly smaller wait times between age of noticing signs of autism and age of receiving the diagnosis compared to Mid and Delayed families, (c) the odds of receiving a later or delayed autism diagnosis were higher on average for caregivers who reported receiving a misdiagnosis compared to caregivers who reported not receiving a misdiagnosis, and (d) the experience of perceived racial barriers was comparable across the Early, Mid, and Delayed families.

Our study mirrors existing knowledge and confirms that the majority of Black youth are receiving an earlier autism diagnosis than what older findings have demonstrated (Constantino et al., 2020; Mandell et al., 2007). The majority of youth in our sample were diagnosed before 4 years of age (72%), while fewer families received a delayed diagnosis (28%). Even though this suggests positive developments in early developmental screening practices (Pierce et al., 2011), challenges persist for Black families of autistic youth who are diagnosed later in life. One clear disparity highlighted in our study was that Delayed families first noticed signs of autism nearly three times later (*M* = 6.86) than Early (*M* = 2.05) and Mid (*M* = 2.97) families. Research findings on factors that contribute to delayed diagnostic timing have been mixed. Some researchers contribute this to differences in parental concerns about child development, while others contribute it to provider incompetencies in identifying autism (Donohue et al., 2019; Weitlauf et al., 2023). In the present study, caregivers were often the first to notice signs of autism (61.4%) followed by their pediatrician (14.9%). This suggests that caregivers are likely the ones to initiate concerns about their child to their providers; thus, if parents do not have concerns, then receiving a diagnosis becomes delayed. Notably, Delayed families received a diagnosis around 6-years of age, which is around the time children start primary school. It is thus conceivable that Delayed families did not raise concerns until issues or challenges in school arose that impacted the child’s daily functioning. It is also possible that delays in noticing first signs of autism may be attributed to differences in symptom severity, parental beliefs, and clinician perspectives. For instance, Black autistic youth without intellectual disabilities are less likely to be identified as autistic than Black autistic children with a cooccurring intellectual disability (Christensen et al., 2016; Constantino et al., 2020; Wiggins et al., 2020). Further, Donohue et al.’s (2019) research provides some evidence that Black caregivers of autistic youth are less likely to report autism specific concerns, including concerns about restricted and repetitive behaviors and social challenges. Weitlauf et al. (2023) provide further evidence that Black caregivers of autistic youth primarily endorse speech, social skill, and behavior concerns. Indeed, nearly half the sample expressed having minimal to no access to educational or community resources. Further, on average, autistic youth in the delayed group had older caregivers than caregivers in the Early and Mid group. This may reflect differences in caregiver knowledge and stigma related to having an autistic child, potentially pointing to a need for greater screening and psychoeducational supports surrounding autism for older parents. This is especially beneficial given the association between parental age and autism related outcomes (Lyall et al., 2020). Nevertheless, the significant gap between the age at which first signs of autism are noticed suggest that greater educational initiatives, campaigns, and community outreach activities are needed to spread knowledge about autism to families of color, community lay members, and professionals who serve diverse populations (Aylward et al., 2021).

The road to receiving an early autism diagnosis is further delayed by the gaps in timing between the age at which first signs of autism are noticed and the age at which a diagnosis is received. In the present study, there was a significant relation between wait times and diagnostic profiles. Specifically, Delayed families waited nearly 2 years to receive an autism diagnosis after first signs of autism were noticed, while Mid and Early families waited only 6 months or less. Although this gap is significant, other studies have estimated the gap to be 3 to 3.5 years, suggesting potential improvements (Crane et al., 2016; Zuckerman et al., 2015). A number of studies have ascribed diagnostic wait gap times to provider-level barriers, including lack of knowledge and confidence in identifying autism (Mazurek et al., 2019; Snijder et al., 2022). Poor provider autism competency is further reflected in the number of providers caregivers interact with until they receive a diagnosis. Among the studies that have examined the experiences of Black families as they seek an autism diagnosis, caregivers report visiting one to more than five providers before obtaining an autism diagnosis (Constantino et al., 2020; Weitlauf et al., 2023). In addition, findings from several studies attest to the fact that a “wait and see” approach is often adopted by providers, which tends to further delay the age at which families receive a diagnosis (Zuckerman et al., 2015). Contrary to the “wait and see” approach, Edwards et al. (2021) identified that providers often adopted a “wait and evaluate further” approach, which also perpetuates delays and may be driven my provider uncertainty due to lack of training. In fact, the study conducted by Edward and colleagues also indicated there was a general agreement among pediatric health care providers that they were not comfortable discussing a potential diagnosis of autism to parents of young children (birth to 5 years of age). The complex intersection of culture, co-occurring challenges, and disability may also exacerbate provider feelings of incompetency diagnosing children with autism earlier.

Our findings also identified misdiagnoses as a significant differential factor for Early, Mid, and Delayed families. In the present study, around 46% of caregivers in the Delayed group received a misdiagnosis, while approximately 17% of families in the Early and Mid-group received a misdiagnosis. This illustrates that the risk of receiving a misdiagnosis is nearly threefold in Black families of autistic youth diagnosed later than life. Indeed, Black families of autistic youth are more likely to be misdiagnosed with disruptive behavior disorders (e.g., conduct disorder and oppositional defiant disorder; Mandell et al., 2007). Research indicates a higher incidence of aggressive behaviors in autistic youth as compared to neurotypical children which may drive these misdiagnoses (Quetsch et al., 2023). This not only delays access to intervention services and optimal care, but it creates tension and stress for caregivers who are trying to find the right supports and services for their child (Crane et al., 2016).

Increased rates of misdiagnoses have also been credited to provider racial bias. In fact, in a study examining the experiences of Black and multiracial families seeking an autism diagnosis, mothers shared that providers were “passing off behaviors that Black children display as ‘a Black thing’” and that they “expect Black kids to naturally be wilder and have behavior problems” (p. 5; Weitlauf et al., 2023). Likewise, white individuals are more likely to implicitly associate pictures of white individuals with autism and pictures of Black people with conduct disorder (Obeid et al., 2021). In its current state, the U.S. workforce currently lacks professionals who are competent in diagnosing, treating, and supporting autistic individuals, especially Black autistic individuals (McBain et al., 2020). Given that the clinical manifestation of autism is often comorbid with other symptoms and diagnoses, greater research and clinical attention should be paid toward creating measures and best practices that include consideration of culture and differential diagnoses to help caregivers and professionals better identify symptoms of autism in individuals with intersectional identities (de Leeuw et al., 2020; Diemer et al., 2022).

The majority of recent studies examining perceptions and experiences of Black caregivers of autistic youth as they navigate the diagnostic service delivery system have highlighted the essential role culture plays in their experiences (Fisher et al., 2022; Onovbiona et al., 2023; Weitlauf et al., 2023). In these studies, Black caregivers of autistic youth report experiencing racism and discrimination, cultural dissonance between themselves and their diagnostic providers, and shame and stigma associated with an autism diagnosis that negatively impacted their diagnostic journey (Onovbiona et al., 2023; Pearson et al., 2020; Pearson & Meadan, 2018; Weitlauf et al., 2023). Given the salient racial and cultural barriers experienced by Black caregivers of autistic youth in prior studies, we predicted that racial barriers would worsen family engagement with services, increase mistrust between families and providers, and would ultimately impede diagnostic progress and lead to delayed diagnostic timing. Despite the high endorsement of provider mistrust, use of unfamiliar medical jargon, low perceived provider knowledge, and ignorance to the realities of racism from providers, no significant associations were found between racial barriers and diagnostic timing. Black caregivers of autistic youth in other studies have expressed concerns such as: “Having a Dr not of my race give me a diagnosis then discuss how costly [care] is didn’t empower me” (p. 5) and “With the Black community it’s hard for us to accept that our child is different” (p. 6; Weitlauf et al., 2023). These findings suggest that cultural and racial barriers may potentially have a greater impact on factors such as family empowerment, perceived support, and child acceptance *after* they receive a diagnosis (Weitlauf et al., 2023). It is also quite possible that caregiver advocacy, which was not formally assessed in this study, played a larger role in helping families overcome the racial barriers they experienced. Advocacy among Black caregivers of autistic youth has been shown to be a powerful tool to help them push through the obstacles that they face throughout their diagnostic journey (Onovbiona et al., 2023). Although no significant relations were observed between perceived racial barriers and diagnostic timing in the present study (Early, Mid, and Delayed families), this does not negate the potential detrimental impact racial barriers can have on the experience, outcomes, and well-being of Black families of autistic youth. Understanding the impact racial barriers have on the quality of care and well-being of Black caregivers of autistic youth can give us critical insight into how to better support families before, during, and after receiving an autism diagnosis.

## Limitations, Clinical Implications, and Future Directions

Although the current study provides an important addition to the literature on factors that impact early identification for Black families of autistic youth, several limitations must be considered. First, the sample used in this study were families directly connected to the SPARK database. Despite its expansive network, SPARK is limited to a national sample of families and autistic individuals who have the resources and interest in engaging in psychological or genetic research. Given that many Black families have stated their distrust in research or medical providers, families participating in this research may be a unique sample and not representative of more distrustful families. Second, individual youth factors (e.g., cognitive scores, symptom ratings) were not collected for this study and thus these factors cannot be explored to determine the impact of diagnostic timing for youth in the sample. This is an important factor to consider as Black autistic youth are nearly twice as likely to be diagnosed with an intellectual disability than their white counterparts (Maenner et al., 2023). It is possible that the presence or absence of intellectual disability may have also played a key role the timing of a child’s diagnosis or experiences of racism. Third, although the present study identifies differences in characteristics between Early, Mid, and Delayed families of autistic youth, we are not necessarily sure *why* this gap exists. For instance, our findings demonstrated that Delayed families have significantly higher rates of misdiagnoses than Early and Mid families, but we cannot conclude the drivers of higher rates of misdiagnoses in Delayed families. Future research should consider potential factors such as provider autism knowledge, parental reports, or provider racial bias. Further, the Racial Barriers Scale used in the study was developed by the researcher and not a psychometrically supported measure, which may impact the validity of the results. Lastly, our study did not include a comparison group (e.g., white autistic youth, Latine autistic youth) and therefore cannot determine if the characteristics that differed between Early, Mid, and Delayed families would be comparable to another racial or ethnic group. This lack of comparison limits our ability to determine if cultural factors and systems-level factors are influencing the differences between diagnostic profiles.

In spite of these limitations, our findings add to a strong and expanding body of literature on factors that contribute to early and delayed identification for Black families of autistic youth. The results reported here have important clinical implications that may guide us in creating trainings for providers, community members, and caregivers. The findings point to high misdiagnosis rates, wait times, and delays in noticing first signs of autism as characteristics that differentiate families diagnosed early and later in life. Taken within the larger body of literature, future applications should include implementing and disseminating trainings for healthcare providers in navigating differential diagnoses and identifying signs of autism. The content for the trainings should include Black families of autistic youth to illuminate their lived experiences while receiving a diagnosis, perceptions surrounding receiving a diagnosis, and interpretations of behaviors and symptoms from both provider and family viewpoints to create a more seamless discussion that avoids jargon, stigma, and distrust. Further efforts should be made to inform caregivers in the community of the importance of early identification, enhance autism awareness, destigmatize autism diagnoses, and spread resources to the larger community. Given the extensive wait list across autism diagnostic services and the lengthy gap between the time at which first autism signs are noticed and the time a diagnosis is received, educators and health care professionals should implement more supports for families while they are navigating long waitlists. For example, interdisciplinary collaborations among parents, primary care providers, therapists, social workers, and additional specialists. Parents Taking Action (PTA), a parent-led program for caregivers of Black children waitlisted for autism or other neurodevelopmental concerns includes story-telling, information on autism, videos on typical development, and stress management skills to support Black caregivers navigating the wait (Dababnah et al., 2023). In addition, given the high proportion of families that endorsed difficulties with health literacy, autism knowledge, and provider autism knowledge, increased advocacy supports should be made available to help reduce the disparities Black families face (Pearson et al., 2022). Finally, the results of this study may point to a need for culturally responsive assessment measures. Like much of the current autism literature, autism assessments are normed from majority samples of white children, devaluing the norms of people with divergent cultural and ethnic backgrounds (Reynolds & Suziki, 2013). Creating assessments that consider sociocultural factors is essential to improve autism identification for culturally diverse autistic youth who may otherwise continue to be misidentified (Harris et al., 2014; Pham & Charles, 2023). For example, recognizing differences in socioeconomic resources, limited eye contact in certain cultures, and caregiver attitudes toward an autism diagnosis (Hussain et al., 2023).

## Conclusion

To date, improvements have been made in early identification of autism; however, challenges in receiving a timely diagnosis remain for some Black autistic youth. Black families who receive delays in an autism diagnosis are more likely to face challenges with misdiagnoses, notice signs of autism later in life, and experience extended wait times between the time they notice first signs and the time in which they receive a diagnosis compared to families diagnosed earlier. In order to improve early identification for Black autistic youth who are at risk for receiving delayed diagnostic care, further research should examine factors and practices that improve autism knowledge among professionals and caregivers (e.g., advocacy supports), enhance differential assessment practices, and integrate culturally responsive practices into autism assessment and screening procedures.

## Footnotes

1 In accordance with the social model and preferences of the larger autism community, the following article uses identity-first language when referring to autistic persons. Alternatively, “autism spectrum disorder” or “autism” is used in this paper when talking about the definition of the diagnostic criteria, prevalence diagnoses, or of the diagnosis itself.
